# Supplementary material for: Sex Affects Human Premature Neonates’ Blood Metabolome According to Gestational Age, Parenteral Nutrition, and Caffeine Treatment
Source: Metabolites. 2021 Mar 9;11(3):158. doi: 10.3390/metabo11030158 (PMC8000935; doi:10.3390/metabo11030158)
Supplement: Supplementary file 1 [file metabolites-11-00158-s001.pdf]

Supplementary Table S1. List of metabolites measured through a targeted metabolomic approach and their abbreviations

Amino acids	Satured acylcarnitines	Hydroxylated acylcarnitines	Unsaturated acylcarnitine
Alanine (Ala)	Free carnitine (C0)	3-Hydroxybutyrylcarnitine (C4OH)	Tiglylcarnitine (C5:1)
Arginine (Arg)	Acetylcarnitine (C2)	3-Hydroxyisovalerylcarnitine/3-hydroxy-2- methylbutyrylcarnitine (C5OH)	Hexenoylcarnitine (C6:1)
Argininosuccinate (ArArgsuc)	Propionylcarnitine (C3)	3-Hydroxyhexanoylcarnitine (C6OH)	Octenoylcarnitine (C8:1)
Aspartate (Asp)	Butyrylcarnitine/isobutyrylcarnitine (C4)	3-Hydroxydodecanoylcarnitine (C12OH)	Decenoylcarnitine (C10:1)
Citrulline (Cit)	Isovalerylcarnitine + methylbutyrylcarnitine (C5)	3-Hydroxytetradecanoylcarnitine (C14OH)	Decadienoylcarnitine (C10:2)
Glutamate (Glu)	Hexanoylcarnitine (C6)	3-Hydroxyhexadecanoylcarnitine (C16OH)	Dodecenoylcarnitine (C12:1)
Glycine (Gly)	Octanoylcarnitine (C8)	3-Hydroxyhexadecenoylcarnitine (C16:1OH)	Tetradecenoylcarnitine (C14:1)
Methionine (Met)	Decanoylcarnitine (C10)	3-hydroxy-octadecanoyl (C18OH)	Tetradecadienoylcarnitine (C14:2)
Ornitine (Orn)	Dodecanoylcarnitine (C12)	3-Hydroxyoctadecenoylcarnitine (C18:1OH)	Hexadecenoylcarnitine (C16:1)
Phenylalanine (Phe)	Tetradecanoylcarnitine (C14)		Octadecenoylcarnitine (C18:1)
Tyrosine (Tyr)	Hexadacanoylcarnitine (C16)		Octadecadienoylcarnitine (C18:2)
Valine (Val)	Octadecanoylcarnitine (C18)		
Xle (Leucine + Isoleucine)	Malonylcarnitine (C3DC)		
	Methylmalonilcarnitine (C4DC)		
	Glutarylcarnitine (C5DC)		
	Methylglutarylcarnitine (C6DC)		
	Octanedioylcarnitine (C8DC)		
	Decanedioylcarnitine (C10DC)		
	Total esterified		
	Esterifed/free carinitine		

Supplementary Table S2. Intra-sex analysis of GA effect

	Female VPI (n= 66)	Female MLPI (n= 71)	p	Male VPI (n= 81)	Male MLPI (n= 93)	p
Ala	130.7 (110.2-158.1)	144.5 (110.0-178.8)	0.13	124.4 (94.8-147.2)	143.2 (110.7-171.0)	0.0008
Val	113.1 (93.6-146.9)	106.9 (89.8-131.9)	0.32	110.8 (87.5-129.1)	106.1 (85.4-128.6)	0.65
Xle	106.3 (94.4-130.6)	106.6 (83.2-122.8)	0.19	106.8 (89.0-123.3)	100.4 (87.6-127.2)	0.42
Met	17.4 (12.9-22.4)	16.0 (12.3-22.4)	0.43	16.6 (13.3-20.4)	17.2 (13.5-21.9)	0.22
Phe	47.8 (41.3-56.0)	44.7 (37.3-53.6)	0.06	45.3 (39.4-52.2)	43.5 (37.6-50.8)	0.13
Tyr	52.0 (42.9-80.8)	42.4 (30.4-60.8)	0.002	45.5 (30.7-72.2)	45.7 (27.4-66.9)	0.51
Asp	21.2 (16.7-29.3)	17.9 (16.5-23.9)	0.08	17.2 (12.9-22.8)	19.1 (14.5-26.8)	0.17
Glu	172.7 (128.7-209.2)	168.9 (135.7-211.9)	0.80	149.4 (117.5-190.2)	165.1 (136.6-215.0)	0.01
Gly	265.2 (214.3-308.8)	266.5 (214.3-310.7)	0.96	246.8 (205.0-273.4)	237.8 (204.1-282.0)	0.86
Orn	29.2 (23.0-42.3)	26.9 (20.1-41.1)	0.29	27.5 (20.6-33.6)	26.2 (21.8-36.5)	0.80
Cit	8.9 (6.6-11.4)	7.3 (5.6-10.4)	0.05	7.4 (5.9-8.9)	6.9 (5.8-8.7)	0.80
Arg	6.6 (4.3-11.3)	6.0 (4.0-9.4)	0.17	6.8 (4.1-9.9)	5.5 (3.0-8.8)	0.10
C0	28.9 (24.5-40.4)	29.3 (22.2-37.9)	0.63	31.6 (24.1-39.6)	32.4 (25.8-42.1)	0.29
C2	20.4 (15.7-26.9)	20.4 (16.0-24.3)	0.77	20.9 (16.7-26.9)	22.2 (18.3-27.7)	0.30
C3	2.5 (1.5-3.4)	2.2 (1.7-2.8)	0.40	3.0 (2.1-3.6)	2.4 (1.6-3.1)	0.002
C4	0.37 (0.26-0.50)	0.26 (0.22-0.39)	0.004	0.36 (0.28-0.58)	0.30 (0.24-0.41)	0.006
C5:1	0.03 (0.02-0.04)	0.03 (0.02-0.03)	0.02	0.03 (0.02-0.04)	0.03 (0.02-0.04)	0.78
C5	0.23 (0.19-0.32)	0.19 (0.14-0.30)	0.03	0.25 (0.20-0.32)	0.20 (0.14)	<0.001
C6	0.05 (0.04-0.06)	0.04 (0.03-0.05)	0.06	0.05 (0.04-0.06)	0.05 (0.04-0.06)	0.43
C5OH	0.12 (0.10-0.16)	0.11 (0.09-0.16)	0.05	0.13 (0.10-0.15)	0.12 (0.09-0.14)	0.11
C8	0.08 (0.06-0.12)	0.07 (0.05-0.10)	0.02	0.09 (0.07-0.13)	0.08 (0.06-0.11)	0.04
C3DC	0.04 (0.03-0.05)	0.04 (0.03-0.05)	0.62	0.04 (0.03-0.05)	0.03 (0.03-0.04)	0.04
C10:1	0.08 (0.05-0.12)	0.07 (0.05-0.09)	0.11	0.07 (0.06-0.11)	0.07 (0.05-0.09)	0.07
C10	0.07 (0.05-0.09)	0.06 (0.05-0.08)	0.29	0.07 (0.05-0.09)	0.07 (0.05-0.08)	0.54
C4DC	0.11 (0.09-0.14)	0.10 (0.08-0.14)	0.48	0.11 (0.09-0.13)	0.12 (0.09-0.16)	0.12
C5DC	0.04 (0.04-0.06)	0.04 (0.03-0.06)	0.30	0.05 (0.04-0.06)	0.04 (0.03-0.05)	0.08
C12:1	0.03 (0.03-0.05)	0.03 (0.03-0.04)	0.57	0.03 (0.02-0.04)	0.03 (0.02-0.05)	0.85
C12	0.10 (0.08-0.14)	0.11 (0.08-0.15)	0.49	0.10 (0.07-0.16)	0.12 (0.08-0.16)	0.36
C6DC	0.03 (0.02-0.04)	0.03 (0.02-0.04)	0.64	0.03 (0.02-0.03)	0.03 (0.02-0.04)	0.68
C14:2	0.06 (0.05-0.07)	0.05 (0.04-0.06)	0.007	0.06 (0.04-0.07)	0.05 (0.04-0.07)	0.22
C14:1	0.11 (0.09-0.13)	0.10 (0.08-0.14)	0.54	0.12 (0.08-0.16)	0.11 (0.08-0.14)	0.62
C14	0.17 (0.13-0.24)	0.17 (0.13-0.21)	0.63	0.18 (0.12-0.26)	0.019 (0.14-0.25)	0.98
C8DC	0.03 (0.02-0.04)	0.03 (0.02-0.03)	0.32	0.03 (0.02-0.04)	0.03 (0.02-0.03)	0.21
C16:1	0.12 (0.09-0.17)	0.12 (0.09-0.18)	0.90	0.14 (0.10-0.21)	0.12 (0.10-0.18)	0.17
C16	1.46 (1.07-2.02)	1.58 (1.34-2.11)	0.23	1.68 (1.14-2.66)	1.91 (1.36-2.46)	0.23
C10DC	0.29 (0.21-0.43)	0.36 (0.23-0.46)	0.15	0.33 (0.25-0.53)	0.37 (0.27-0.51)	0.51
C16OH	0.02 (0.02-0.03)	0.02 (0.01-0.03)	0.18	0.02 (0.02-0.03)	0.02 (0.02-0.03)	0.38
C18:1	1.19 (0.87-1.48)	1.17 (0.97-1.36)	0.91	0.23 (0.95-1.56)	1.24 (1.05-1.56)	0.42
C18	0.73 (0.53-0.99)	0.72 (0.54-0.88)	0.57	0.80 (0.59-0.99)	0.74 (0.63-1.02)	0.83
C18:1OH	0.02 (0.02-0.03)	0.02 (0.02-0.03)	0.15	0.02 (0.02-0.03)	0.02 (0.02-0.03)	0.77
C4-OH	0.08 (0.06-0.11)	0.07 (0.06-0.10)	0.03	0.09 (0.07-0.11)	0.08 (0.06-0.10)	0.01
C6OH	0.03 (0.02-0.04)	0.03 (0.02-0.04)	0.09	0.03 (0.02-0.04)	0.03 (0.03-0.04)	0.20
C6:1	0.05 (0.04-0.06)	0.05 (0.04-0.07)	0.40	0.05 (0.04-0.07)	0.05 (0.04-0.06)	0.49
C8:1	0.10 (0.07-0.15)	0.08 (0.06-0.11)	0.05	0.10 (0.06-0.15)	0.08 (0.06-0.11)	0.07
C10:2	0.04 (0.03-0.05)	0.03 (0.03-0.04)	0.06	0.04 (0.03-0.05)	0.03 (0.03-0.04)	0.02

C12-OH	0.02 (0.02-0.03)	0.02 (0.02-0.03)	0.36	0.02 (0.02-0.02)	0.02 (0.02-0.02)	0.59
C14-OH	0.02 (0.02-0.03)	0.02 (0.02-0.03)	0.70	0.02 (0.02-0.03)	0.02 (0.02-0.03)	0.34
C16:1OH	0.04 (0.03-0.05)	0.04 (0.03-0.05)	0.92	0.04 (0.03-0.05)	0.04 (0.03-0.06)	0.45
C18OH	0.02 (0.01-0.02)	0.01 (0.01-0.02)	0.19	0.01 (0.01-0.02)	0.01 (0.01-0.02)	0.08
C18:2	0.33 (0.20-0.49)	0.28 (0.17-0.35)	0.10	0.30 (0.21-0.49)	0.35 (0.24-0.44)	0.87
Esterified	29.0 (23.5-38.8)	29.2 (24.0-35.4)	0.58	30.5 (26.3-38.3)	31.1 (27.0-38.3)	0.49
Esterified/Free	0.95 (0.82-1.15)	0.94 (0.81-1.18)	0.90	0.99 (0.87-1.15)	0.94 (0.79-1.08)	0.16
Argsuc	0.13 (0.09-0.22)	0.13 (0.09-0.21)	0.83	0.12 (0.10-0.17)	0.12 (0.10-0.20)	0.99

Supplementary Table S3. Inter-sex analysis of GA effect

	Female VPI (n= 66)	Male VPI (n= 81)	p	Female MLPI (n= 71)	Male MLPI (n= 93)	p
Ala	130.7 (110.2-158.1)	124.4 (94.8-147.2)	0.03	144.5 (110.0-178.8)	143.2 (110.7-171.0)	0.84
Val	113.1 (93.6-146.9)	110.8 (87.5-129.1)	0.60	106.9 (89.8-131.9)	106.1 (85.4-128.6)	0.91
Xle	106.3 (94.4-130.6)	106.8 (89.0-123.3)	0.60	106.6 (83.2-122.8)	100.4 (87.6-127.2)	0.63
Met	17.4 (12.9-22.4)	16.6 (13.3-20.4)	0.28	16.0 (12.3-22.4)	17.2 (13.5-21.9)	0.33
Phe	47.8 (41.3-56.0)	45.3 (39.4-52.2)	0.15	44.7 (37.3-53.6)	43.5 (37.6-50.8)	0.65
Tyr	52.0 (42.9-80.8)	45.5 (30.7-72.2)	0.03	42.4 (30.4-60.8)	45.7 (27.4-66.9)	0.97
Asp	21.2 (16.7-29.3)	17.2 (12.9-22.8)	0.002	17.9 (16.5-23.9)	19.1 (14.5-26.8)	0.91
Glu	172.7 (128.7-209.2)	149.4 (117.5-190.2)	0.04	168.9 (135.7-211.9)	165.1 (136.6-215.0)	0.92
Gly	265.2 (214.3-308.8)	246.8 (205.0-273.4)	0.01	266.5 (214.3-310.7)	237.8 (204.1-282.0)	0.05
Orn	29.2 (23.0-42.3)	27.5 (20.6-33.6)	0.13	26.9 (20.1-41.1)	26.2 (21.8-36.5)	0.96
Cit	8.9 (6.6-11.4)	7.4 (5.9-8.9)	0.005	7.3 (5.6-10.4)	6.9 (5.8-8.7)	0.68
Arg	6.6 (4.3-11.3)	6.8 (4.1-9.9)	0.44	6.0 (4.0-9.4)	5.5 (3.0-8.8)	0.47
C0	28.9 (24.5-40.4)	31.6 (24.1-39.6)	0.55	29.3 (22.2-37.9)	32.4 (25.8-42.1)	0.05
C2	20.4 (15.7-26.9)	20.9 (16.7-26.9)	0.63	20.4 (16.0-24.3)	22.2 (18.3-27.7)	0.04
C3	2.5 (1.5-3.4)	3.0 (2.1-3.6)	0.05	2.2 (1.7-2.8)	2.4 (1.6-3.1)	0.72
C4	0.37 (0.26-0.50)	0.36 (0.28-0.58)	0.73	0.26 (0.22-0.39)	0.30 (0.24-0.41)	0.13
C5:1	0.03 (0.02-0.04)	0.03 (0.02-0.04)	0.57	0.03 (0.02-0.03)	0.03 (0.02-0.04)	0.08
C5	0.23 (0.19-0.32)	0.25 (0.20-0.32)	0.37	0.19 (0.14-0.30)	0.20 (0.14)	0.68
C6	0.05 (0.04-0.06)	0.05 (0.04-0.06)	0.39	0.04 (0.03-0.05)	0.05 (0.04-0.06)	0.03
C5OH	0.12 (0.10-0.16)	0.13 (0.10-0.15)	0.86	0.11 (0.09-0.16)	0.12 (0.09-0.14)	0.62
C8	0.08 (0.06-0.12)	0.09 (0.07-0.13)	0.24	0.07 (0.05-0.10)	0.08 (0.06-0.11)	0.07
C3DC	0.04 (0.03-0.05)	0.04 (0.03-0.05)	0.44	0.04 (0.03-0.05)	0.03 (0.03-0.04)	0.12
C10:1	0.08 (0.05-0.12)	0.07 (0.06-0.11)	0.87	0.07 (0.05-0.09)	0.07 (0.05-0.09)	0.99
C10	0.07 (0.05-0.09)	0.07 (0.05-0.09)	0.90	0.06 (0.05-0.08)	0.07 (0.05-0.08)	0.51
C4DC	0.11 (0.09-0.14)	0.11 (0.09-0.13)	0.97	0.10 (0.08-0.14)	0.12 (0.09-0.16)	0.02
C5DC	0.04 (0.04-0.06)	0.05 (0.04-0.06)	0.53	0.04 (0.03-0.06)	0.04 (0.03-0.05)	0.87
C12:1	0.03 (0.03-0.05)	0.03 (0.02-0.04)	0.65	0.03 (0.03-0.04)	0.03 (0.02-0.05)	0.97
C12	0.10 (0.08-0.14)	0.10 (0.07-0.16)	0.81	0.11 (0.08-0.15)	0.12 (0.08-0.16)	0.61
C6DC	0.03 (0.02-0.04)	0.03 (0.02-0.03)	0.53	0.03 (0.02-0.04)	0.03 (0.02-0.04)	0.29
C14:2	0.06 (0.05-0.07)	0.06 (0.04-0.07)	0.59	0.05 (0.04-0.06)	0.05 (0.04-0.07)	0.35
C14:1	0.11 (0.09-0.13)	0.12 (0.08-0.16)	0.52	0.10 (0.08-0.14)	0.11 (0.08-0.14)	0.38
C14	0.17 (0.13-0.24)	0.18 (0.12-0.26)	0.46	0.17 (0.13-0.21)	0.019 (0.14-0.25)	0.10
C8DC	0.03 (0.02-0.04)	0.03 (0.02-0.04)	0.89	0.03 (0.02-0.03)	0.03 (0.02-0.03)	0.94
C16:1	0.12 (0.09-0.17)	0.14 (0.10-0.21)	0.08	0.12 (0.09-0.18)	0.12 (0.10-0.18)	0.62
C16	1.46 (1.07-2.02)	1.68 (1.14-2.66)	0.18	1.58 (1.34-2.11)	1.91 (1.36-2.46)	0.07
C10DC	0.29 (0.21-0.43)	0.33 (0.25-0.53)	0.05	0.36 (0.23-0.46)	0.37 (0.27-0.51)	0.20

C16OH	0.02 (0.02-0.03)	0.02 (0.02-0.03)	0.91	0.02 (0.01-0.03)	0.02 (0.02-0.03)	0.59
C18:1	1.19 (0.87-1.48)	0.23 (0.95-1.56)	0.39	1.17 (0.97-1.36)	1.24 (1.05-1.56)	0.04
C18	0.73 (0.53-0.99)	0.80 (0.59-0.99)	0.38	0.72 (0.54-0.88)	0.74 (0.63-1.02)	0.10
C18:1OH	0.02 (0.02-0.03)	0.02 (0.02-0.03)	0.40	0.02 (0.02-0.03)	0.02 (0.02-0.03)	0.48
C4-OH	0.08 (0.06-0.11)	0.09 (0.07-0.11)	0.59	0.07 (0.06-0.10)	0.08 (0.06-0.10)	0.42
C6OH	0.03 (0.02-0.04)	0.03 (0.02-0.04)	0.99	0.03 (0.02-0.04)	0.03 (0.03-0.04)	0.33
C6:1	0.05 (0.04-0.06)	0.05 (0.04-0.07)	0.53	0.05 (0.04-0.07)	0.05 (0.04-0.06)	0.28
C8:1	0.10 (0.07-0.15)	0.10 (0.06-0.15)	0.91	0.08 (0.06-0.11)	0.08 (0.06-0.11)	0.89
C10:2	0.04 (0.03-0.05)	0.04 (0.03-0.05)	0.99	0.03 (0.03-0.04)	0.03 (0.03-0.04)	0.42
C12-OH	0.02 (0.02-0.03)	0.02 (0.02-0.02)	0.57	0.02 (0.02-0.03)	0.02 (0.02-0.02)	0.93
C14-OH	0.02 (0.02-0.03)	0.02 (0.02-0.03)	0.38	0.02 (0.02-0.03)	0.02 (0.02-0.03)	0.65
C16:1OH	0.04 (0.03-0.05)	0.04 (0.03-0.05)	0.35	0.04 (0.03-0.05)	0.04 (0.03-0.06)	0.07
C18OH	0.02 (0.01-0.02)	0.01 (0.01-0.02)	0.23	0.01 (0.01-0.02)	0.01 (0.01-0.02)	0.22
C18:2	0.33 (0.20-0.49)	0.30 (0.21-0.49)	0.54	0.28 (0.17-0.35)	0.35 (0.24-0.44)	0.004
Esterified	29.0 (23.5-38.8)	30.5 (26.3-38.3)	0.45	29.2 (24.0-35.4)	31.1 (27.0-38.3)	0.03
Esterified/Free	0.95 (0.82-1.15)	0.99 (0.87-1.15)	0.62	0.94 (0.81-1.18)	0.94 (0.79-1.08)	0.53
Argsuc	0.13 (0.09-0.22)	0.12 (0.10-0.17)	0.46	0.13 (0.09-0.21)	0.12 (0.10-0.20)	0.65

Supplementary Table S4. Intra-sex analysis of PN effect in females

	Female VPI (n= 66)			Female MLPI (n= 71)		
	**NPN (n= 28)**	**PN (n= 38)**	**p**	**No PN (n= 28)**	**PN (n= 38)**	**p**
Ala	135.7 (115.1-153.7)	129.8 (109.0-160.1)	0.66	142.5 (117.6-171.4)	148.2 (110.0-180.1)	0.64
Val	111.4 (91.0-149.7)	114.3 (93.6-142.3)	0.81	108.8 (73.6-146.0)	105.5 (92.1-121.4)	0.66
Xle	108.0 (98.6-137.7)	104.1 (91.9-125.4)	0.31	103.3 (76.1-131.1)	107.3 (84.4-118.2)	0.57
Met	16.5 (11.4-25.2)	17.5 (13.3-21.7)	0.95	13.8 (9.5-20.4)	16.3 (13.6-23.3)	0.07
Phe	45.4 (40.9-54.7)	49.4 (42.7-56.4)	0.26	44.2 (33.1-54.1)	46.8 (40.0-53.1)	0.25
Tyr	53.0 (44.4-81.4)	52 (41.4-79.8)	0.69	48.3 (30.0-64.4)	38.5 (33.8-47.8)	0.32
Asp	26.2 (20.0-40.7)	19.4 (14.9-24.6)	0.002	20.1 (16.9-33.3)	17.8 (16.5-20.6)	0.11
Glu	206.6 (172.7-256.8)	150.5 (123.8-176.0)	0.0003	196.1 (142.8-261.3)	156.9 (135.7-174.3)	0.007
Gly	283.2 (240.4-332.3)	260 (201.2-285.5)	0.01	266.0 (197.5-323.4)	266.5 (243.2-291.5)	0.90
Orn	36.4 (22.2-45.9)	28.1 (23.7-35.0)	0.13	23.7 (17.7-37.4)	30.2 (22.1-41.1)	0.15
Cit	10.5 (8.8-12.6)	7.0 (6.3-9.7)	0.01	7.3 (5.6-11.3)	6.9 (5.8-9.7)	0.59
Arg	8.8 (5.3-12.3)	6.0 (4.0-9.5)	0.06	5.4 (2.5-9.3)	6.0 (4.3-9.4)	0.36
C0	28.5 (24.4-39.6)	29.6 (24.5-41.1)	0.47	33.5 (23.7-40.3)	26.2 (22.2-37.1)	0.26
C2	20.6 (17.8-26.4)	19.8 (15.1-27.0)	0.47	21.9 (16.6-24.9)	20.4 (15.6-23.2)	0.23
C3	2.5 (1.3-3.8)	2.5 (1.5-3.2)	0.95	2.0 (1.7-2.7)	2.4 (1.7-3.0)	0.23
C4	0.39 (0.26-0.51)	0.36 (0.28-0.50)	0.88	0.26 (0.23-0.34)	0.29 (0.22-0.41)	0.43
C5:1	0.03 (0.02-0.04)	0.03 (0.02-0.04)	0.51	0.03 (0.02-0.03)	0.03 (0.02-0.04)	0.13
C5	0.22 (0.17-0.33)	0.23 (0.19-0.32)	0.64	0.02 (0.01-0.02)	0.23 (0.17-0.33)	0.005
C6	0.05 (0.04-0.06)	0.04 (0.04-0.05)	0.31	0.04 (0.03-0.05)	0.04 (0.03-0.05)	0.54
C5OH	0.14 (0.12-0.17)	0.11 (0.09-0.15)	0.02	0.11 (0.09-0.17)	0.10 (0.09-0.14)	0.77
C8	0.08 (0.07-0.11)	0.08 (0.06-0.12)	0.89	0.07 (0.05-0.08)	0.07 (0.05-0.10)	0.26
C3DC	0.04 (0.03-0.06)	0.04 (0.02-0.04)	0.58	0.04 (0.02-0.04)	0.04 (0.03-0.05)	0.08
C10:1	0.08 (0.06-0.11)	0.08 (0.05-0.14)	0.95	0.07 (0.05-0.08)	0.06 (0.05-0.09)	0.88
C10	0.07 (0.05-0.10)	0.07 (0.05-0.08)	0.57	0.07 (0.05-0.08)	0.06 (0.05-0.08)	0.72
C4DC	0.12 (0.10-0.16)	0.10 (0.08-0.14)	0.007	0.12 (0.08-0.14)	0.10 (0.08-0.12)	0.06

C5DC	0.05 (0.04-0.07)	0.04 (0.03-0.05)	0.17	0.04 (0.03-0.06)	0.04 (0.04-0.05)	0.82
C12:1	0.04 (0.03-0.05)	0.03 (0.02-0.04)	0.02	0.03 (0.02-0.04)	0.03 (0.03-0.05)	0.41
C12	0.11 (0.09-0.14)	0.10 (0.07-0.14)	0.32	0.09 (0.07-0.11)	0.14 (0.11-0.18)	<0.0001
C6DC	0.02 (0.02-0.03)	0.03 (0.02-0.04)	0.25	0.02 (0.02-0.03)	0.04 (0.03-0.04)	<0.0001
C14:2	0.05 (0.05-0.06)	0.06 (0.05-0.08)	0.15	0.05 (0.04-0.06)	0.05 (0.04-0.06)	0.28
C14:1	0.11 (0.09-0.13)	0.11 (0.08-0.13)	0.78	0.10 (0.07-0.12)	0.11 (0.09-0.14)	0.04
C14	0.18 (0.15-0.24)	0.17 (0.11-0.25)	0.32	0.15 (0.12-0.20)	0.18 (0.15-0.21)	0.11
C8DC	0.03 (0.02-0.04)	0.03 (0.02-0.03)	0.10	0.03 (0.02-0.03)	0.03 (0.2-0.03)	0.005
C16:1	0.13 (0.10-0.21)	0.11 (0.07-0.15)	0.14	0.12 (0.10-0.18)	0.12 (0.09-0.18)	0.66
C16	1.46 (1.19-2.07)	1.44 (0.96-1.99)	0.49	1.57 (1.33-2.21)	1.62 (1.38-2.08)	0.63
C10DC	0.30 (0.24-0.45)	0.27 (0.20-0.41)	0.26	0.32 (0.22-0.47)	0.39 (0.24-0.43)	0.23
C16OH	0.02 (0.02-0.03)	0.02 (0.02-0.03)	0.30	0.02 (0.01-0.03)	0.02 (0.02-0.03)	0.12
C18:1	1.20 (0.93-1.47)	0.18 (0.80-1.48)	0.59	1.16 (0.97-1.36)	1.22 (0.88-1.33)	0.82
C18	0.71 (0.49-0.89)	0.74 (0.56-1.00)	0.47	0.65 (0.51-0.84)	0.75 (0.65-0.96)	0.21
C18:1OH	0.02 (0.02-0.03)	0.02 (0.02-0.03)	0.74	0.02 (0.02-0.03)	0.02 (0.02-0.03)	0.97
C4-OH	0.08 (0.06-0.10)	0.09 (0.07-0.11)	0.56	0.07 (0.05-0.10)	0.07 (0.06-0.09)	0.83
C6OH	0.04 (0.03-0.05)	0.03 (0.02-0.04)	0.10	0.03 (0.02-0.04)	0.03 (0.03-0.04)	0.85
C6:1	0.05 (0.04-0.07)	0.05 (0.04-0.06)	0.43	0.05 (0.03-0.07)	0.05 (0.04-0.07)	0.29
C8:1	0.09 (0.08-0.14)	0.10 (0.06-0.16)	0.96	0.08 (0.05-0.11)	0.08 (0.06-0.11)	0.87
C10:2	0.04 (0.03-0.05)	0.04 (0.03-0.05)	0.34	0.03 (0.02-0.04)	0.04 (0.03-0.05)	0.02
C12-OH	0.02 (0.02-0.03)	0.02 (0.02-0.02)	0.08	0.02 (0.01-0.02)	0.02 (0.02-0.03)	0.32
C14-OH	0.02 (0.02-0.03)	0.02 (0.02-0.02)	0.17	0.02 (0.02-0.03)	0.02 (0.02-0.03)	0.83
C16:1OH	0.04 (0.03-0.05)	0.04 (0.03-0.05)	0.77	0.04 (0.03-0.05)	0.04 (0.03-0.05)	0.28
C18OH	0.02 (0.01-0.02)	0.01 (0.01-0.02)	0.04	0.01 (0.01-0.02)	0.02 (0.01-0.02)	0.59
C18:2	0.22 (0.17-0.37)	0.38 (0.23-0.52)	0.006	0.27 (0.19-0.31)	0.03 (0.02-0.04)	0.65
Esterified	31.2 (24.6-38.2)	28.5 (22.7-39.6)	0.52	30.6 (24.4-35.4)	28.2 (23.7-34.8)	0.55
Esterified/Free	1.01 (0.86-1.32)	0.93 (0.82-1.11)	0.12	0.93 (0.77-1.18)	0.97 (0.87-1.17)	0.34
Argsuc	0.21 (0.13-0.34)	0.10 (0.08-0.13)	<0.0001	0.15 (0.11-0.26)	0.12 (0.09-0.15)	0.11

Supplementary Table S5. Intra-sex analysis of PN effect in males

	Male VPI (n= 81)			Male MLPI (n= 93)		
	NPN (n= 30)	PN (n= 51)	p	NPN (n= 49)	PN (n= 44)	p
Ala	106.5 (94.3-139.8)	128.4 (94.8-147.2)	0.48	138.4 (110.3-186.3)	147.0 (113.1-161.1)	0.84
Val	109.2 (84.4-128.7)	111.2 (87.5-129.2)	0.87	114.2 (77.8-161.0)	104.9 (92.3-120.0)	0.42
Xle	94.9 (86.4-117.3)	109.3 (96.2-137.8)	0.08	112.4 (81.7-131.4)	96.1 (89.6-119.1)	0.52
Met	14.6 (11.4-18.0)	17.3 (13.3-21.1)	0.17	17.0 (12.9-22.6)	17.7 (13.8-21.1)	0.81
Phe	40.6 (38.7-51.2)	48.9 (40.1-54.1)	0.03	42.5 (33.0-49.7)	45.6 (38.5-51.7)	0.08
Tyr	44.4 (33.4-63.6)	47.8 (29.9-78.7)	0.55	47.3 (33.9-68.9)	40.0 (23.5-63.5)	0.14
Asp	18.0 (12.9-23.9)	16.9 (12.9-21.7)	0.48	19.9 (16.2-30.7)	17.4 (12.2-23.6)	0.04
Glu	162.0 (140.6-199.0)	143.5 (112.0-190.2)	0.05	191.6 (149.9-258.2)	149.0 (124.7-189.7)	0.005
Gly	235.8 (179.3-276.7)	249.3 (208.6-273.4)	0.32	245.6 (194.2-282.0)	234.8 (207.9-288.9)	0.85
Orn	24.1 (18.9-33.4)	28.0 (21.0-35.8)	0.42	25.9 (20.4-39.1)	26.2 (22.1-33.7)	0.95
Cit	7.8 (5.9-9.3)	7.1 (5.9-8.6)	0.57	6.9 (5.8-9.3)	7.0 (5.9-8.6)	0.93
Arg	5.8 (3.8-9.0)	7.3 (4.1-10.1)	0.53	5.3 (2.9-10.3)	5.6 (3.3-8.0)	0.88
C0	31.0 (26.4-39.0)	32.6 (23.7-40.0)	0.70	32.2 (25.7-41.0)	34.2 (26.1-43.5)	0.58
C2	22.8 (19.4-31.3)	20.0 (15.2-25.4)	0.01	22.2 (19.2-27.9)	22.2 (18.2-27.3)	0.63
C3	3.0 (2.2-3.8)	2.8 (2.0-3.6)	0.58	2.1 (1.4-3.1)	2.5 (1.8-3.1)	0.22
C4	0.35 (0.29-0.53)	0.36 (0.27-0.58)	0.95	0.32 (0.3-0.43)	0.30 (0.24-0.39)	0.49
C5:1	0.03 (0.02-0.04)	0.03 (0.02-0.04)	0.61	0.03 (0.02-0.04)	0.03 (0.02-0.04)	0.38
C5	0.23 (0.18-0.35)	0.25 (0.20-0.32)	0.67	0.17 (0.13-0.26)	0.22 (0.18-0.25)	0.03
C6	0.05 (0.04-0.06)	0.05 (0.04-0.06)	0.64	0.05 (0.03-0.06)	0.05 (0.04-0.06)	0.79
C5OH	0.14 (0.10-0.17)	0.12 (0.10-0.14)	0.15	0.12 (0.10-0.16)	0.11 (0.09-0.13)	0.13
C8	0.10 (0.08-0.14)	0.08 (0.06-0.11)	0.04	0.07 (0.06-0.10)	0.09 (0.06-0.11)	0.35
C3DC	0.04 (0.03-0.05)	0.04 (0.03-0.05)	0.79	0.03 (0.03-0.04)	0.04 (0.03-0.04)	0.06
C10:1	0.09 (0.05-0.15)	0.07 (0.06-0.10)	0.19	0.06 (0.05-0.08)	0.08 (0.06-0.10)	0.02
C10	0.08 (0.06-0.12)	0.06 (0.05-0.08)	0.06	0.06 (0.04-0.08)	0.07 (0.06-0.09)	0.007
C4DC	0.12 (0.09-0.16)	0.11 (0.09-0.13)	0.12	0.13 (0.10-0.16)	0.12 (0.09-0.15)	0.18
C5DC	0.05 (0.04-0.07)	0.04 (0.04-0.06)	0.09	0.05 (0.03-0.05)	0.04 (0.03-0.06)	0.85
C12:1	0.04 (0.02-0.06)	0.03 (0.02-0.04)	0.29	0.03 (0.03-0.04)	0.04 (0.02-0.05)	0.68
C12	0.12 (0.07-0.16)	0.10 (0.07-0.14)	0.40	0.10 (0.07-0.12)	0.15 (0.11-0.22)	<0.0001
C6DC	0.02 (0.02-0.04)	0.03 (0.02-0.03)	0.42	0.02 (0.02-0.03)	0.03 (0.02-0.04)	0.0004
C14:2	0.06 (0.05-0.07)	0.06 (0.04-0.08)	0.99	0.05 (0.04-0.07)	0.05 (0.05-0.06)	0.58
C14:1	0.13 (0.08-0.16)	0.11 (0.08-0.14)	0.15	0.10 (0.08-0.12)	0.12 (0.10-0.16)	0.002
C14	0.24 (0.14-0.30)	0.15 (0.12-0.25)	0.05	0.17 (0.13-0.21)	0.20 (0.15-0.26)	0.07
C8DC	0.03 (0.02-0.04)	0.03 (0.02-0.04)	0.43	0.03 (0.02-0.03)	0.03 (0.02-0.04)	0.13
C16:1	0.17 (0.11-0.26)	0.13 (0.09-0.18)	0.08	0.12 (0.09-0.18)	0.12 (0.10-0.18)	0.98
C16	1.81 (1.44-2.85)	1.50 (1.07-2.26)	0.12	0.97 (036-2.39)	1.87 (1.39-2.53)	0.83
C10DC	0.35 (0.26-0.57)	0.33 (0.23-0.48)	0.39	0.35 (0.26-0.49)	0.39 (0.28-0.53)	0.36
C16OH	0.02 (0.02-0.03)	0.02 (0.02-0.03)	0.90	0.02 (0.02-0.03)	0.02 (0.02-0.03)	0.98
C18:1	1.23 (1.01-1.54)	1.26 (0.89-1.67)	0.64	1.22 (1.00-1.53)	1.29 (1.08-1.57)	0.80
C18	0.84 (0.59-1.06)	0.79 (0.58-0.97)	0.59	0.69 (0.64-0.91)	0.85 (0.61-1.11)	0.19
C18:1OH	0.02 (0.02-0.03)	0.02 (0.02-0.03)	0.47	0.02 (0.02-0.03)	0.02 (0.02-0.03)	0.91
C4-OH	0.09 (0.07-0.11)	0.08 (0.07-0.10)	0.28	0.08 (0.06-0.09)	0.08 (0.06-0.10)	0.61
C6OH	0.03 (0.02-0.04)	0.04 (0.02-0.04)	0.97	0.03 (0.03-0.04)	0.03 (0.03-0.04)	0.53
C6:1	0.05 (0.04-0.07)	0.05 (0.04-0.07)	0.83	0.05 (0.04-0.06)	0.05 (0.04-0.06)	0.74
C8:1	0.12 (0.07-0.17)	0.08 (0.06-0.13)	0.08	0.08 (0.06-0.10)	0.08 (0.06-0.13)	0.14
C10:2	0.05 (0.03-0.06)	0.04 (0.03-0.05)	0.02	0.03 (0.03-0.04)	0.03 (0.02-0.04)	0.41

C12-OH	0.02 (0.02-0.04)	0.02 (0.02-0.02)	0.26	0.02 (0.01-0.02)	0.02 (0.02-0.03)	0.34
C14-OH	0.02 (0.02-0.04)	0.02 (0.02-0.03)	0.02	0.02 (0.01-0.03)	0.02 (0.02-0.03)	0.06
C16:1OH	0.04 (0.03-0.05)	0.05 (0.03-0.05)	0.57	0.04 (0.03-0.05)	0.05 (0.04-0.06)	0.11
C18OH	0.02 (0.01-0.02)	0.01 (0.01-0.02)	0.55	0.01 (0.01-0.02)	0.01 (0.01-0.02)	0.08
C18:2	0.29 (0.21-0.49)	0.34 (0.24-0.53)	0.34	0.30 (0.19-0.41)	0.38 (0.29-0.47)	0.07
Esterified	32.9 (28.1-44.6)	28.5 (22.6-35.5)	0.03	31.1 (27.1-40.1)	31.6 (27.0-36.8)	0.95
Esterified/ Free	1.12 (0.93-1.37)	0.93 (0.80-1.03)	0.001	0.99 (0.82-1.12)	0.92 (0.79-1.04)	0.18
Argsuc	0.15 (0.10-0.19)	0.11 (0.10-0.16)	0.34	0.16 (0.10-0.28)	0.11 (0.08-0.13)	0.01

Supplementary Table S6. Inter-sex analysis of PN effect

	PN (n= 170)			NPN (n=141)		
	**Females (n= 75)**	**Males (n= 95)**	**p**	**Females (n= 62)**	**Males (n= 79)**	**p**
Ala	139.4 (109.8-171.0)	139.8 (100.8-155.8)	0.20	141.4 (115.3-168.1)	130.7 (100.3-165.0)	0.34
Val	111.0 (92.1-125.9)	105.0 (88.9-124.2)	0.58	110.0 (87.9-146.9)	111.9 (80.3-149.7)	0.82
Xle	106.7 (90.5-118.7)	104.2 (91.6-123.3)	0.90	106.1 (83.6-134.5)	101.7 (85.1-129.8)	0.73
Met	17.1 (13.3-22.2)	17.5 (13.7-21.1)	0.81	15.2 (10.2-22.6)	16.1 (12.8-21.6)	0.74
Phe	47.8 (41.0-56.0)	47.0 (39.5-52.4)	0.56	44.9 (36.6-54.1)	41.4 (36.7-50.5)	0.22
Tyr	44.7 (35.6-70.7)	45.4 (25.9-70.4)	0.31	50.4 (38.0-76.6)	46.2 (33.6-68.2)	0.23
Asp	17.9 (15.8-23.0)	17.0 (12.4-23.4)	0.17	22.9 (17.2-39.2)	19.7 (14.9-27.3)	0.05
Glu	155.0 (129.1-176.0)	144.1 (114.2-190.2)	0.33	205.8 (164.5-259.5)	173.8 (146.0-227.6)	0.11
Gly	262.9 (214.3-289.9)	242.6 (208.0-273.4)	0.11	281.5 (223.7-330.7)	245.3 (184.5-279.7)	0.004
Orn	29.0 (22.6-37.4)	27.4 (21.8-34.2)	0.40	28.1 (19.9-44.6)	24.8 (19.9-37.4)	0.40
Cit	6.9 (6.0-9.7)	7.1 (5.9-8.6)	0.46	9.3 (6.2-11.7)	7.4 (5.8-9.3)	0.02
Arg	6.0 (4.1-9.5)	6.5 (3.7-9.0)	0.69	7.5 (4.1-10.9)	5.7 (3.2-9.5)	0.29
C0	28.2 (23.7-37.9)	32.6 (25.2-42.4)	0.10	30.3 (23.7-39.9)	31.6 (25.8-40.7)	0.44
C2	20.3 (15.5-24.6)	21.0 (16.7-25.9)	0.38	21.4 (17.6-26.0)	22.5 (19.4-29.9)	0.10
C3	2.5 (1.6-3.1)	2.7 (1.95-3.39)	0.15	2.1 (1.6-3.3)	2.6 (1.6-3.5)	0.26
C4	0.32 (0.25-0.49)	0.32 (0.25-0.49)	0.81	0.3 (0.2-0.5)	0.33 (0.25-0.48)	0.21
C5:1	0.03 (0.02-0.04)	0.03 (0.02-0.04)	0.83	0.03 (0.02-0.03)	0.03 (0.02-0.04)	0.13
C5	0.23 (0.18-0.32)	0.23 (0.20-0.27)	0.72	0.19 (0.14-0.26)	0.20 (0.14-0.27)	0.57
C6	0.04 (0.03-0.05)	0.05 (0.04-0.06)	0.13	0.04 (0.03-0.06)	0.05 (0.03-0.06)	0.22
C5OH	0.11 (0.09-0.15)	0.12 (0.10-0.14)	0.63	0.13 (0.10-0.17	0.13 (0.10-0.17)	0.96
C8	0.07 (0.06-0.12)	0.08 (0.06-0.11)	0.47	0.08 (0.06-0.09)	0.09 (0.07-0.12	0.04
C3DC	0.04 (0.03-0.05)	0.04 (0.03-0.05)	0.74	0.04 (0.03-0.05)	0.03 (0.03-0.04)	0.65
C10:1	0.07 (0.05-0.11)	0.07 (0.06-0.10)	0.61	0.07 (0.06-0.10)	0.07 (0.05-0.10)	0.53
C10	0.06 (0.05-0.08)	0.07 (0.05-0.09)	0.42	0.07 (0.05-0.08)	0.06 (0.05-0.09)	0.99
C4DC	0.10 (0.08-0.14)	0.11 (0.09-0.14)	0.05	0.12 (0.10-0.15)	0.13 (0.09-0.16)	0.49
C5DC	0.04 (0.03-0.05)	0.04 (0.03-0.06)	0.79	0.05 (0.04-0.06)	0.05 (0.04-0.06)	0.57
C12:1	0.03 (0.02-0.04)	0.03 (0.02-0.04)	0.99	0.04 (0.03-0.05)	0.03 (0.02-0.05)	0.65
C12	0.12 (0.09-0.16)	0.12 (0.08-0.19)	0.75	0.10 (0.08-0.13)	0.10 (0.07-0.14)	0.47
C6DC	0.03 (0.02-0.04)	0.03 (0.02-0.04)	0.07	0.02 (0.02-0.03)	0.02 (0.02-0.03)	0.74
C14:2	0.05 (0.04-0.07)	0.06 (0.04-0.07)	0.66	0.05 (0.04-0.06)	0.05 (0.04-0.07)	0.36
C14:1	0.11 (0.08-0.14)	0.12 (0.08-0.15)	0.50	0.10 (0.08-0.13)	0.11 (0.08-0.14)	0.38
C14	0.18 (0.12-0.21)	0.19 (0.13-0.25)	0.27	0.16 (0.13-0.21)	0.18 (0.13-0.26)	0.21
C8DC	0.03 (0.02-0.03)	0.03 (0.02-0.04)	1.00	0.03 (0.02-0.03)	0.03 (0.02-0.04)	0.82
C16:1	0.11 (0.09-0.17)	0.12 (0.09-0.18)	0.19	0.13 (0.10-0.19)	0.14 (0.10-0.20)	0.43
C16	1.55 (1.10-2.04)	1.69 (1.15-2.48)	0.25	1.55 (1.21-2.12)	1.93 (1.39-2.57)	0.03

C10DC	0.34 (0.23-0.43)	0.36 (0.25-0.53)	0.12	0.31 (0.23-0.45)	0.35 (0.26-0.50)	0.14
C16OH	0.02 (0.02-0.03)	0.02 (0.02-0.03)	0.65	0.02 (0.01-0.03)	0.02 (0.02-0.03)	0.72
C18:1	1.22 (0.86-1.45)	1.27 (0.96-1.59)	0.10	1.17 (0.95-1.44)	1.22 (1.01-1.54)	0.26
C18	0.74 (0.56-1.00)	0.81 (0.61-1.05)	0.31	0.69 (0.50-0.84)	0.69 (0.62-0.97)	0.06
C18:1OH	0.02 (0.02-0.03)	0.02 (0.02-0.03)	0.79	0.02 (0.02-0.03)	0.02 (0.02-0.03)	0.90
C4-OH	0.08 (0.06-0.11)	0.08 (0.07-0.10)	0.62	0.08 (0.06-0.10)	0.08 (0.06-0.10)	0.52
C6OH	0.03 (0.02-0.04)	0.03 (0.02-0.04)	0.24	0.03 (0.02-0.04)	0.03 (0.02-0.04)	0.63
C6:1	0.05 (0.04-0.06)	0.05 (0.04-0.07)	0.66	0.05 (0.03-0.07)	0.05 (0.04-0.06)	0.97
C8:1	0.08 (0.06-0.14)	0.08 (0.06-0.13)	1.00	0.09 (0.06-0.13)	0.09 (0.06-0.13)	0.99
C10:2	0.04 (0.03-0.05)	0.03 (0.03-0.05)	0.11	0.03 (0.03-0.05)	0.04 (0.03-0.05)	0.28
C12-OH	0.02 (0.02-0.03)	0.02 (0.02-0.02)	0.91	0.02 (0.02-0.03)	0.02 (0.02-0.03)	0.58
C14-OH	0.02 (0.02-0.03)	0.02 (0.02-0.03)	0.63	0.02 (0.02-0.03)	0.02 (0.02-0.03)	0.28
C16:1OH	0.04 (0.03-0.05)	0.05 (0.03-0.06)	0.14	0.04 (0.03-0.05)	0.04 (0.03-0.05)	0.18
C18OH	0.02 (0.01-0.02)	0.01 (0.01-0.02)	0.08	0.02 (0.01-0.02)	0.01 (0.01-0.02)	0.31
C18:2	0.32 (0.21-0.46)	0.36 (0.25-0.49)	0.24	0.25 (0.17-0.33)	0.30 (0.21-0.44)	0.03
Esterified	28.4 (23.1-36.3)	31.0 (25.7-36.5)	0.26	30.6 (24.4-36.5)	31.5 (27.5-43.4)	0.07
Esterified/Free	0.94 (0.83-1.15)	0.93 (0.79-1.03)	0.20	0.95 (0.81-1.27)	1.03 (0.86-1.19)	0.31
Argsuc	0.11 (0.08-0.14)	0.11 (0.09-0.14)	0.89	0.19 (0.11-0.32)	0.14 (0.10-0.26)	0.07

Supplementary Table S7. Intra-sex analysis of caffeine effect in females

	**Females VPI (n= 66)**			**Females MLPI (n= 71)**		
	**No caffeine (n= 41)**	**Caffeine (n= 25)**	**p**	**No caffeine (n= 55)**	**Caffeine (n= 16)**	**p**
Ala	148.1 (110.2-168.0)	123.7 (109.8-146.2)	0.18	148.2 (109.8-178.8)	138.7 (126.9-179.9)	0.79
Val	113.7 (98.0-131.1)	110.5 (89.5-152.6)	0.60	106.8 (85.1-131.9)	110.3 (99.77-127.9)	0.55
Xle	106.6 (90.5-129.8)	106.0 (96.5-131.8)	0.68	104.7 (83.2-127.9)	107.9 (92.9-114.1)	0.79
Met	17.6 (13.7-22.6)	15.6 (12.1-21.8)	0.34	16.3 (12.5-23.3)	15.3 (11.6-18.5)	0.29
Phe	49.5 (44.3-60.4)	45.0 (39.2-52.4)	0.14	45.1 (35.9-55.2)	43.7 (41.5-53.1)	0.97
Tyr	51.5 (43.7-81.7)	57.3 (41.4-73.8)	0.89	39.6 (32.5-64.4)	45.6 (28.5-59.1)	0.76
Asp	23.3 (17.7-30.7)	20.0 (14.4-24.1)	0.10	17.7 (15.8-23.7)	20.7 (17.6-29.0)	0.14
Glu	180.8 (144.9-226.2)	149.0 (123.8-179.2)	0.03	168.6 (134.9-195.9)	188.2 (143.2-260.5)	0.24
Gly	280.1 (235.6-325.3)	260.7 (213.3-282.6)	0.07	259.0 (210.5-308.3)	288.2 (233.4-316.2)	0.52
Orn	29.7 (24.0-44.3)	28.0 (21.2-40.5)	0.31	26.3 (20.4-37.4)	29.8 (19.6-41.7)	0.95
Cit	8.8 (6.8-11.4)	9.0 (5.9-11.3)	0.48	7.3 (5.5-10.0)	7.7 (6.5-10.9)	0.52
Arg	6.6 (4.3-11.6)	6.5 (4.7-9.1)	0.83	5.5 (3.9-10.2)	7.8 (5.0-9.2)	0.29
C0	28.5 (24.5-44.8)	29.8 (25.5-35.8)	0.67	28.2 (20.4-37.9)	33.6 (26.1-42.0)	0.12
C2	20.4 (15.5-27.0)	19.3 (16.6-24.5)	0.89	19.8 (15.6-23.4)	22.2 (19.6-24.8)	0.18
C3	2.37 (1.46-3.31)	2.51 (1.95-3.86)	0.46	2.1 (1.7-2.7)	2.4 (1.9-3.3)	0.35
C4	0.39 (0.26-0.50)	0.35 (0.28-0.47)	0.66	0.26 (0.22-0.37)	0.27 (0.24-0.40)	0.76
C5:1	0.03 (0.03-0.04)	0.03 (0.02-0.03)	0.02	0.03 (0.02-0.04)	0.03 (0.02-0.03)	0.94
C5	0.23 (0.19-0.31)	0.23 (0.199-0.33)	0.57	0.19 (0.14-0.30)	0.22 (0.15-0.30)	0.64
C6	0.05 (0.04-0.06)	0.04 (0.03-0.06)	0.46	0.04 (0.03-0.05)	0.04 (0.03-0.05)	0.96
C5OH	0.12 (0.10-0.17)	0.13 (0.11-0.15)	0.69	0.10 (0.09-0.16)	0.11 (0.10-0.14)	0.94
C8	0.08 (0.06-0.10)	0.09 (0.07-0.13)	0.34	0.07 (0.05-0.09)	0.08 (0.05-0.10)	0.67
C3DC	0.04 (0.03-0.04)	0.04 (0.03-0.05)	0.57	0.04 (0.03-0.05)	0.04 (0.02-0.04)	0.12
C10:1	0.08 (0.05-0.12)	0.08 (0.06-0.11)	0.81	0.06 (0.05-0.09)	0.08 (0.06-0.09)	0.16
C10	0.06 (0.05-0.08)	0.07 (0.05-0.09)	0.60	0.06 (0.05-0.08)	0.07 (0.05-0.08)	0.49

C4DC	0.12 (0.09-0.15)	0.10 (0.09-0.13)	0.25	0.10 (0.08-0.14)	0.11 (0.08-0.14)	0.65
C5DC	0.04 (0.04-0.06)	0.04 (0.03-0.05)	0.25	0.04 (0.03-0.06)	0.04 (0.03-0.05)	0.43
C12:1	0.03 (0.03-0.05)	0.03 (0.03-0.04)	0.62	0.03 (0.03-0.04)	0.04 (0.03-0.05)	0.37
C12	0.11 (0.08-0.14)	0.10 (0.07-0.13)	0.31	0.11 (0.08-0.15)	0.11 (0.09-0.15)	0.91
C6DC	0.03 (0.02-0.04)	0.03 (0.02-0.04)	0.98	0.03 (0.02-0.04)	0.03 (0.02-0.04)	0.52
C14:2	0.06 (0.05-0.07)	0.06 (0.05-0.07)	0.85	0.05 (0.04-0.06)	0.05 (0.04-0.07)	0.54
C14:1	0.11 (0.09-0.15)	0.11 (0.08-0.12)	0.46	0.10 (0.07-0.14)	0.10 (0.09-0.14)	0.65
C14	0.17 (0.13-0.25)	0.16 (0.14-0.21)	0.88	0.17 (0.13-0.21)	0.17 (0.13-0.20)	0.86
C8DC	0.03 (0.02-0.04)	0.03 (0.02-0.03)	0.15	0.03 (0.02-0.03)	0.03 (0.02-0.03)	0.36
C16:1	0.12 (0.09-0.22)	0.11 (0.09-0.13)	0.19	0.12 (0.09-0.17)	0.11 (0.08-0.21)	0.79
C16	1.47 (1.01-2.37)	11.45 (1.23-1.81)	0.97	1.59 (1.20-2.04)	1.57 (1.41-2.38)	0.44
C10DC	0.32 (0.22-0.46)	0.28 (0.21-0.36)	0.35	0.35 (0.23-0.43)	0.39 (0.28-0.46)	0.59
C16OH	0.02 (0.02-0.03)	0.02 (0.02-0.02)	0.07	0.02 (0.01-0.03)	0.02 (0.02-0.03)	0.81
C18:1	1.22 (0.88-1.54)	1.14 (0.80-1.34)	0.34	1.17 (0.97-1.33)	0.17 (1.01-1.40)	0.97
C18	0.74 (0.51-0.96)	0.70 (0.56-1.05)	0.80	0.74 (0.51-0.89)	0.68 (0.61-0.83)	0.87
C18:1OH	0.02 (0.02-0.03)	0.02 (0.02-0.03)	0.65	0.02 (0.02-0.03)	0.02 (0.02-0.03)	0.68
C4-OH	0.09 (0.06-0.11)	0.08 (0.07-0.11)	0.98	0.07 (0.05-0.10)	0.08 (0.06-0.11)	0.37
C6OH	0.04 (0.03-0.04)	0.03 (0.02-0.04)	0.22	0.03 (0.02-0.04)	0.03 (0.03-0.04)	0.73
C6:1	0.05 (0.03-0.06)	0.05 (0.04-0.06)	0.96	0.05 (0.04-0.07)	0.04 (0.03-0.07)	0.20
C8:1	0.09 (0.06-0.15)	0.11 (0.07-0.16)	0.41	0.08 (0.06-0.11)	0.10 (0.06-0.12)	0.52
C10:2	0.04 (0.03-0.05)	0.04 (0.03-0.05)	0.73	0.03 (0.03-0.04)	0.04 (0.03-0.04)	0.94
C12-OH	0.02 (0.02-0.03)	0.02 (0.02-0.03)	0.99	0.02 (0.02-0.03)	0.02 (0.02-0.03)	0.87
C14-OH	0.02 (0.02-0.03)	0.02 (0.02-0.02)	0.56	0.02 (0.02-0.03)	0.02 (0.02-0.03)	0.55
C16:1OH	0.04 (0.03-0.05)	0.04 (0.03-0.05)	0.37	0.04 (0.03-0.05)	0.04 (0.03-0.04)	0.39
C18OH	0.02 (0.01-0.02)	0.02 (0.01-0.02)	0.95	0.01 (0.01-0.02)	0.02 (0.01-0.02)	0.37
C18:2	0.31 (0.20-0.50)	0.34 (0.20-0.46)	0.97	0.28 (0.16-0.35)	0.28 (0.22-0.34)	0.48
Esterified	29.3 (23.4-39.6)	28.3 (24.0-35.1)	0.85	27.8 (23.1-35.5)	31.4 (27.5-35.1)	0.21
Esterified/Free	0.95 (0.85-1.12)	0.95 (0.82-1.15)	0.62	0.96 (0.83-1.18)	0.93 (0.79-1.00)	0.50
Argsuc	0.14 (0.09-0.25)	0.12 (0.10-0.17)	0.34	0.12 (0.09-0.19)	0.15 (0.10-0.23)	0.24

Supplementary Table S8. Intra-sex analysis of caffeine effect in males

	**Males very preterm (n= 81)**			**Males moderate or late preterm (n= 93)**		
	**No caffeine (n= 52)**	**Caffeine (n= 29)**	**p**	**No caffeine (n= 72)**	**Caffeine (n= 21)**	**p**
Ala	123.1 (91.4-145.3)	126.5 (100.9-148.5)	0.30	145.0 (123.8-173.3)	124.2 (107.1-160.0)	0.38
Val	96.1 (81.7-128.9)	118.2 (111.2-131.0)	0.009	106.1 (82.1-122.6)	112.2 (97.0-170.7)	0.27
Xle	97.9 (85.8-117.5)	112.8 (102.3-137.8)	0.03	96.8 (87.3-125.2)	109.3 (90.8-130.4)	0.60
Met	16.7 (12.0-19.9)	15.6 (13.5-21.5)	0.57	17.3 (13.7-21.5)	17.2 (12.6-22.5)	0.76
Phe	45.9 (38.8-52.2)	45.0 (40.1-54.6)	0.50	43.2 (37.9-49.6)	43.9 (33.5-55.7)	0.79
Tyr	47.2 (30.1-70.6)	43.6 (30.9-73.5)	0.62	47.0 (27.0-65.7)	44.0 (32.2-69.6)	0.75
Asp	16.2 (12.4-22.7)	19.6 (16.4-23.7)	0.16	19.9 (15.5-28.2)	15.4 (12.5-19.3)	0.01
Glu	145.9 (114.1-193.1)	153.2 (133.6-177.8)	0.59	171.3 (136.6-216.5)	156.1 (136.7-199.0)	0.40
Gly	246.6 (203.0-276.8)	249.3 (208.6-269.7)	0.92	246.1 (209.3-308.4)	210.8 (190.1-251.3)	0.03
Orn	25.3 (20.2-32.9)	29.0 (22.7-37.3)	0.13	25.3 (21.7-39.2)	29.5 (22.7-36.4)	0.74
Cit	7.3 (5.7-8.5)	7.6 (6.4-9.3)	0.26	6.9 (5.9-8.5)	7.5 (5.8-11.0)	0.57
Arg	6.1 (3.5-8.6)	8.0 (5.4-11.3)	0.09	5.2 (2.9-8.3)	6.5 (4.0-11.7)	0.19

C0	30.7 (23.8-41.4)	34.2 (26.4-39.0)	0.70	32.5 (25.8-41.1)	31.7 (25.9-60.7)	0.49
C2	20.1 (16.3-26.6)	23.4 (19.4-27.2)	0.16	22.4 (18.5-27.7)	21.3 (17.5-29.0)	0.83
C3	2.86 (1.96-3.53)	3.02 (2.30-3.88)	0.23	2.10 (1.41-3.00)	2.79 (2.22-3.74)	0.02
C4	0.36 (0.27-0.54)	0.39 (0.28-0.58)	0.57	0.30 (0.24-0.38)	0.38 (0.24-0.49)	0.11
C5:1	0.03 (0.02-0.04)	0.03 (0.02-0.04)	0.68	0.03 (0.02-0.04)	0.03 (0.03-0.04)	0.76
C5	0.24 (0.19-0.34)	0.25 (0.21-0.27)	0.98	0.20 (0.14-0.24)	0.25 (0.18-0.37)	0.03
C6	0.05 (0.03-0.06)	0.06 (0.04-0.06)	0.09	0.05 (0.04-0.06)	0.05 (0.03-0.06)	0.66
C5OH	0.12 (0.10-0.15)	0.13 (0.11-0.16)	0.19	0.11 (0.09-0.13)	0.15 (0.13-0.17)	0.0005
C8	0.08 (0.06-0.11)	0.11 (0.08-0.14)	0.04	0.08 (0.06-0.10)	0.09 (0.07-0.11)	0.20
C3DC	0.04 (0.03-0.05)	0.04 (0.03-0.05)	0.82	0.03 (0.03-0.04)	0.04 (0.03-0.05)	0.36
C10:1	0.08 (0.06-0.11)	0.07 (0.05-0.12)	0.49	0.07 (0.05-0.09)	0.06 (0.05-0.13)	0.39
C10	0.7 (0.05-0.09)	0.06 (0.05-0.09)	0.55	0.07 (0.05-0.08)	0.06 (0.05-0.10)	0.81
C4DC	0.11 (0.08-0.13)	0.13 (0.09-0.16)	0.04	0.13 (0.10-0.16)	0.12 (0.09-0.14)	0.19
C5DC	0.05 (0.04-0.06)	0.05 (0.04-0.06)	0.88	0.04 (0.03-0.05)	0.05 (0.04-0.07)	0.20
C12:1	0.03 (0.02-0.05)	0.03 (0.02-0.04)	0.99	0.03 (0.02-0.04)	0.03 (0.02-0.06)	0.74
C12	0.10 (0.08-0.16)	0.10 (0.07-0.14)	0.61	0.12 (0.09-0.16)	0.10 (0.08-0.14)	0.44
C6DC	0.03 (0.02-0.04)	0.02 (0.02-0.03)	0.06	0.02 (0.02-0.04)	0.03 (0.02-0.04)	0.80
C14:2	0.06 (0.04-0.08)	0.06 (0.04-0.07)	0.77	0.05 (0.04-0.06)	0.06 (0.05-0.09)	0.01
C14:1	0.12 (0.08-0.15)	0.12 (0.09-0.16)	0.55	0.11 (0.08-0.14)	0.12 (0.09-0.16)	0.37
C14	0.16 (0.13-0.26)	0.19 (0.12-0.26)	0.75	0.19 (0.14-0.25)	0.17 (0.13-0.24)	0.88
C8DC	0.03 (0.02-0.04)	0.03 (0.02-0.04)	0.26	0.03 (0.02-0.03)	0.03 (0.02-0.04)	0.49
C16:1	0.14 (0.10-0.22)	0.15 (0.10-0.21)	0.79	0.12 (0.10-0.18)	0.12 (0.09-0.19)	0.84
C16	1.71 (1.00-2.62)	1.66 (1.39-2.70)	0.58	1.82 (1.41-2.47)	2.10 (1.08-2.37)	0.74
C10DC	0.34 (0.24-0.57)	0.33 (0.26-0.48)	0.94	0.38 (0.28-0.51)	0.31 (0.20-0.51)	0.34
C16OH	0.02 (0.02-0.03)	0.02 (0.02-0.02)	0.11	0.02 (0.02-0.03)	0.02 (0.02-0.27)	0.65
C18:1	0.18 (0.84-1.66)	1.33 (0.08-1.54)	0.22	1.21 (1.05-1.49)	1.52 (1.08-1.74)	0.11
C18	0.78 (0.54-1.01)	0.89 (0.63-0.99)	0.22	0.72 (0.61-0.97)	0.82 (0.64-1.14)	0.19
C18:1OH	0.02 (0.02-0.03)	0.02 (0.02-0.03)	0.62	0.02 (0.02-0.03)	0.02 (0.02-0.04)	0.28
C4-OH	0.09 (0.07-0.10)	0.09 (0.07-0.11)	0.73	0.08 (0.06-0.09)	0.10 (0.07-0.11)	0.04
C6OH	0.03 (002-0.04)	0.04 (0.0-0.04)	0.38	0.03 (0.03-0.04)	0.03 (0.03-0.04)	0.25
C6:1	0.05 (0.04-0.07)	0.05 (0.04-0.07)	0.88	0.05 (0.04-0.06)	0.06 (0.04-0.07)	0.05
C8:1	0.11 (0.06-0.15)	0.08 (0.06-0.15)	0.54	0.08 (0.06-0.11)	0.10 (0.07-0.15)	0.04
C10:2	0.04 (0.03-0.06)	0.03 (0.03-0.05)	0.08	0.03 (0.02-0.04)	0.03 (0.03-0.04)	0.57
C12-OH	0.02 (0.02-0.03)	0.02 (0.02-0.02)	0.70	0.02 (0.02-0.02)	0.02 (0.02-0.03)	0.70
C14-OH	0.02 (0.2-0.03)	0.02 (0.02-0.03)	0.09	0.02 (0.02-0.03)	0.02 (0.02-0.03)	0.29
C16:1OH	0.04 (0.03-0.05)	0.04 (0.03-0.05)	1.00	0.04 (0.03-0.06)	0.05 (0.03-0.06)	0.82
C18OH	0.01 (0.01-0.02)	0.01 (0.1-0.02)	0.18	0.01 (0.01-0.02)	0.01 (0.01-0.02)	0.69
C18:2	0.30 (0.22-0.50)	0.30 (0.20-0.49)	0.76	0.32 (0.20-0.42)	0.43 (0.33-0.60)	0.01
Esterified	28.4 (25.1-37.8)	33.1 (29.2-38.3)	0.11	31.1 (27.4 -37.2)	30.7 (26.7-44.6)	0.73
Esterified/Free	0.98 (0.86-1.14)	0.99 (0.90-1.19)	0.35	0.95 (0.79-1.10)	0.93 (0.81-1.04)	0.48
Argsuc	0.11 (0.10-0.16)	0.12 (0.11-0.26)	0.24	0.11 (0.09-0.20)	0.13 (0.10-0.19)	0.56

Supplementary Table S9. Inter-sex analysis of caffeine effect

	No caffeine (n= 220)			Caffeine (n= 91)		
	**Females (n= 96)**	**Males (n= 124)**	**p**	**Females (n= 41)**	**Males (n= 50)**	**p**
Ala	148.1 (110.0-172.0)	138.3 (98.0-157.6)	0.11	127.8 (114.9-155.0)	126.5 (101.7-155.8)	0.51
Val	110.7 (90.1-131.7)	103.4 (81.7-125.8)	0.23	110.5 (92.8-143.2)	118.2 (103.0-157.6)	0.13
Xle	105.1 (83.7-128.5)	97.5 (87.0-122.0)	0.71	107.3 (96.5-124.6)	112.2 (98.0-131.5)	0.47
Met	17.4 (12.8-23.0)	16.9 (13.4-20.7)	0.42	15.3 (11.8-21.7)	17.0 (13.3-22.2)	0.21
Phe	47.4 (39.5-56.1)	44.2 (38.4-51.4)	0.09	44.8 (40.9-53.1)	44.5 (39.4-55.4)	0.85
Tyr	46.0 (35.8-73.6)	47.0 (27.6-67.9)	0.37	50.1 (38.0-66.3)	43.6 (30.9-72.2)	0.27
Asp	19.5 (16.6-27.6)	18.6 (13.9-26.2)	0.14	20.2 (17.0-24.7)	17.3 (13.2-21.7)	0.07
Glu	174.8 (135.3-211.9)	159.7 (130.0-208.7)	0.25	163.6 (127.4-207.1)	155.3 (134.5-190.2)	0.58
Gly	266.5 (216.5-320.2)	246.6 (206.5-280.1)	0.02	263.8 (214.3-291.5)	226.2 (199.9-265.2)	0.03
Orn	28.9 (21.5-44.0)	25.3 (20.5-35.5)	0.12	28.3 (19.9-40.9)	29.0 (22.7-37.3)	0.86
Cit	7.6 (6.2-10.8)	7.0 (5.9-8.5)	0.04	8.6 (6.2-11.3)	7.5 (6.0-9.6)	0.53
Arg	6.0 (4.0-11.0)	5.7 (3.2-8.5)	0.16	7.5 (4.7-9.1)	7.1 (4.9-11.5)	0.66
C0	28.4 (22.4-40.6)	32.1 (25.6-41.1)	0.10	31.4 (25.9-37.4)	33.3 (26.0-40.8)	0.33
C2	20.2 (15.5-26.2)	21.6 (17.5-27.1)	0.15	21.1 (18.0-24.8)	22.2 (18.2-27.2)	0.22
C3	2.2 (1.6-3.0)	2.4 (1.7-3.3)	0.29	2.5 (2.0-3.4)	2.8 (2.3-8.9)	0.07
C4	0.30 (0.24-0.49)	0.31 (0.25-0.46)	0.64	0.34 (0.25-0.44)	0.39 (0.26-0.57)	0.14
C5:1	0.03 (0.02-0.04)	0.03 (0.02-0.04)	0.98	0.03 (0.02-0.03)	0.03 (0.02-0.04)	0.21
C5	0.21 (0.15-0.30)	0.21 (0.17-0.27)	0.91	0.23 (0.18-0.32)	0.25 (0.20-0.28)	0.51
C6	0.04 (0.03-0.06)	0.05 (0.04-0.06)	0.39	0.04 (0.03-0.05)	0.05 (0.04-0.06)	0.02
C5OH	0.11 (0.09-0.16)	0.11 (0.10-0.14)	0.54	0.12 (0.10-0.15)	0.14 (0.11-0.16)	0.08
C8	0.07 (0.06-0.10)	0.08 (0.06-0.11)	0.20	0.08 (0.06-0.11)	0.09 (0.07-0.13)	0.10
C3DC	0.04 (0.02-0.05)	0.04 (0.03-0.04)	0.25	0.04 (0.03-0.04)	0.04 (0.03-0.05)	0.49
C10:1	0.07 (0.05-0.10)	0.07 (0.05-0.10)	0.72	0.08 (0.06-0.09)	0.07 (0.05-0.13)	0.63
C10	0.06 (0.05-0.08)	0.07 (0.05-0.09)	0.38	0.07 (0.05-0.08)	0.06 (0.05-0.10)	0.80
C4DC	0.11 (0.09-0.14)	0.12 (0.09-0.15)	0.36	0.10 (0.08-0.13)	0.12 (0.09-0.15)	0.05
C5DC	0.04 (0.04-0.06)	0.04 (0.03-0.06)	0.59	0.04 (0.03-0.05)	0.05 (0.04-0.06)	0.08
C12:1	0.03 (0.03-0.05)	0.03 (0.02-0.05)	0.78	0.04 (0.03-0.04)	0.03 (0.02-0.04)	0.88
C12	0.11 (0.08-0.15)	0.12 (0.08-0.16)	0.61	0.10 (0.08-0.13)	0.10 (0.07-0.14)	0.88
C6DC	0.03 (0.02-0.04)	0.03 (0.02-0.04)	0.44	0.03 (0.02-0.04)	0.02 (0.02-0.03)	0.46
C14:2	0.05 (0.04-0.07)	0.05 (0.04-0.07)	0.95	0.05 (0.04-0.07)	0.06 (0.05-0.08)	0.50
C14:1	0.11 (0.08-0.14)	0.11 (0.08-0.14)	0.68	0.11 (0.09-0.12)	0.12 (0.09-0.16)	0.18
C14	0.07 (0.13-0.22)	0.18 (0.13-0.24)	0.16	0.17 (0.13-0.20)	0.18 (0.12-0.26)	0.34
C8DC	0.03 (0.02-0.04)	0.03 (0.02-0.04)	0.62	0.03 (0.02-0.03)	0.03 (0.02-0.04)	0.44
C16:1	0.12 (0.09-0.18)	0.13 (0.09-0.18)	0.49	0.11 (0.09-0.15)	0.13 (0.10-0.20)	0.08
C16	1.56 (1.08-2.06)	1.67 (1.21-2.38)	0.06	1.53 (1.35-2.04)	1.77 (1.21-2.60)	0.21
C10DC	0.34 (0.23-0.45)	0.35 (0.24-0.49)	0.06	0.30 (0.11-0.41)	0.32 (0.26-0.49)	0.26
C16OH	0.02 (0.02-0.03)	0.02 (0.02-0.03)	0.88	0.02 (0.02-0.02)	0.02 (0.02-0.03)	0.85
C18:1	1.19 (0.91-1.46)	1.20 (0.95-1.48)	0.48	1.16 (0.93-1.34)	1.37 (1.08-1.67)	0.005
C18	0.74 (0.51-0.93)	0.74 (0.55-0.96)	0.27	0.70 (0.60-0.88)	0.86 (0.64-1.08)	0.05
C18:1OH	0.02 (0.02-0.03)	0.02 (0.02-0.03)	0.59	0.02 (0.02-0.03)	0.02 (0.02-0.03)	0.48
C4-OH	0.08 (0.06-0.10)	0.08 (0.06-0.10)	0.67	0.08 (0.07-0.11)	0.09 (0.07-0.11)	0.33
C6OH	0.03 (0.02-0.04)	0.03 (0.02-0.04)	0.73	0.03 (0.02-0.04)	0.04 (0.03-0.04)	0.10
C6:1	0.05 (0.04-0.07)	0.05 (0.04-0.06)	0.25	0.05 (0.04-0.06)	0.05 (0.04-0.07)	0.21
C8:1	0.08 (0.06-0.13)	0.08 (0.06-0.13)	0.83	0.11 (0.07-0.14)	0.09 (0.06-0.15)	0.87
C10:2	0.04 (0.03-0.05)	0.04 (0.03-0.05)	0.85	0.04 (0.03-0.05)	0.03 (0.03-0.05)	0.52
C12-OH	0.02 (0.02-0.03)	0.02 (0.02-0.03)	0.75	0.02 (0.02-0.03)	0.02 (0.02-0.02)	0.68
C14-OH	0.02 (0.02-0.03)	0.02 (0.02-0.03)	0.88	0.02 (0.02-0.03)	0.02 (0.02-0.03)	0.69
C16:1OH	0.04 (0.03-0.05)	0.04 (0.03-0.05)	0.27	0.04 (0.03-0.05)	0.04 (0.03-0.05)	0.05
C18OH	0.01 (0.01-0.02)	0.01 (0.01-0.02)	0.38	0.02 (0.01-0.02)	0.01 (0.01-0.02)	0.06

C18:2	0.28 (0.18-0.40)	0.30 (0.20-0.44)	0.06	0.29 (0.21-0.44)	0.35 (0.27-0.53)	0.18
Esterified	28.3 (23.2-37.6)	30.5 (26.4-37.2)	0.12	30.9 (24.8-35.1)	33.1 (28.1-43.5)	0.09
Esterified/Free	0.96 (0.83-1.17)	0.95 (0.80-1.11)	0.73	0.94 (0.82-1.14)	0.98 (0.86-1.10)	0.75
Argsuc	0.13 (0.09-0.22)	0.12 (0.09-0.19)	0.31	0.13 (0.10-0.21)	0.13 (0.10-0.23)	0.85

Supplementary Table S10. Characteristics of the cohorts stratified by sex and GA

**Pre-term infants [311]**			
**Females [137]**		**Males [174]**	
**VPI [66]**	**MLPI [71]**	**VPI [81]**	**MLPI [93]**
39.4 % caffeine	22.5 % caffeine	35.8 % caffeine	22.6 % caffeine
57.6% PN	53.5% PN	63% PN	47.8% PN
89.4% antibiotics	74.6% antibiotics *	77.8% antibiotics	77.2% antibiotics

Penicillin and aminoglycosides were the most prescribed antibiotics (>90%). * p = 0.03. In squared brackets the number of samples

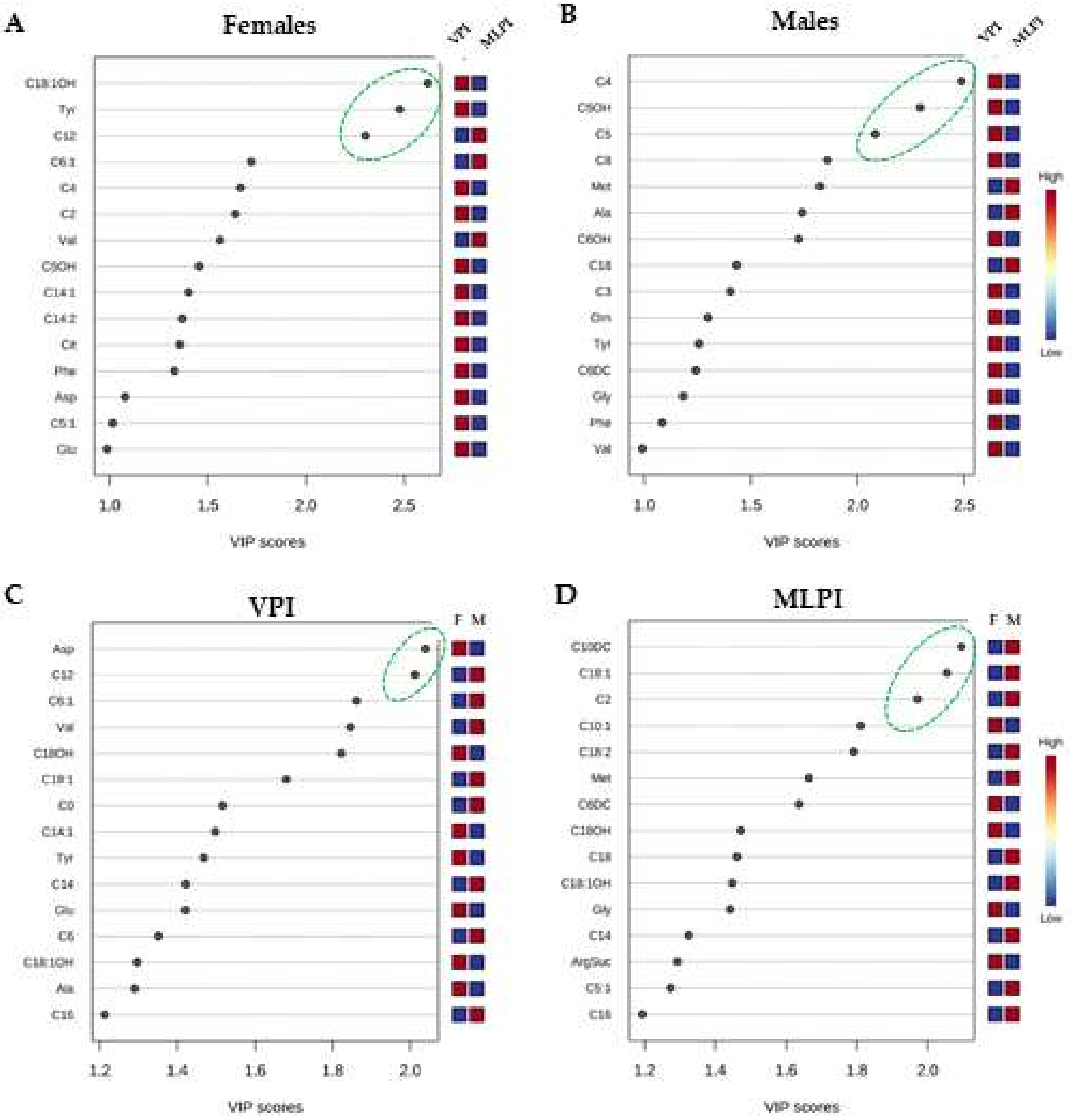

Supplementary Figure S1. Metabolites which differentiate VPI and MLPI groups in female (**A)** and male (B) cohorts, respectively; Discriminant metabolites between VPI (C) and MLPI (D) females and males. Each panel reported the top 15 important discriminant features identified by PLS-DA according to the VIP score. The concentrations of the metabolites were log(2) transformed, and Pareto scaled. The intensity of the colored boxes indicates the relative metabolite abundance in each group

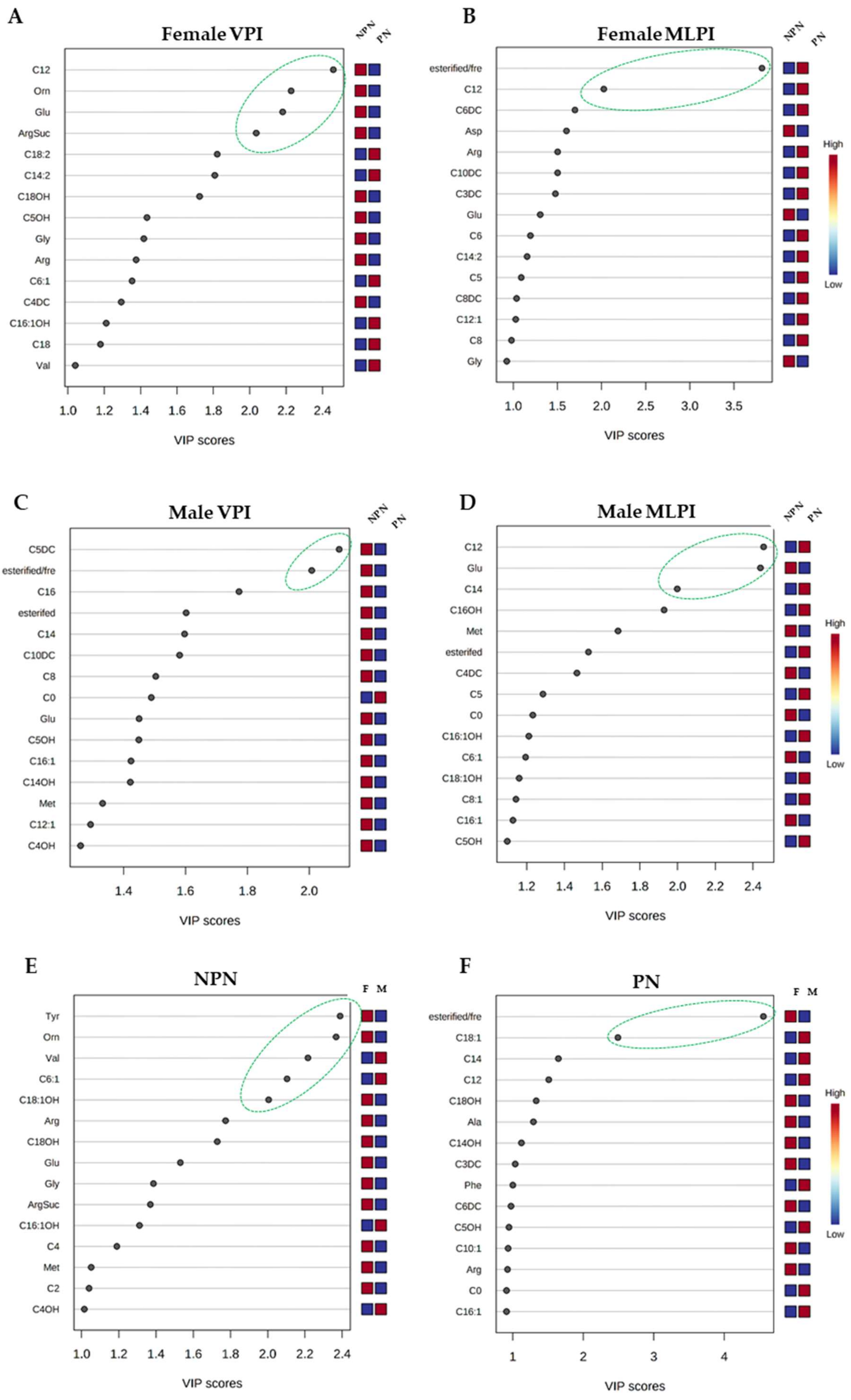
A
Female VPI
NPN
PN
C12
Orn
Glu
ArgSuc
C18:2
C14:2
C18OH
C5OH
Gly
Arg
C6:1
C4DC
C16:1OH
C18
Val
1.0 1.2 1.4 1.6 1.8 2.0 2.2 2.4
VIP scores
B
Female MLPI
NPN
PN
esterified/fre
C12
C6DC
Asp
Arg
C10DC
C3DC
Glu
C6
C14:2
C5
C8DC
C12:1
C8
Gly
High
Low
1.0 1.5 2.0 2.5 3.0 3.5
VIP scores
C
Male VPI
NPN
PN
C5DC
esterified/fre
C16
esterifed
C14
C10DC
C8
C0
Glu
C5OH
C16:1
C14OH
Met
C12:1
C4OH
1.4 1.6 1.8 2.0
VIP scores
D
Male MLPI
NPN
PN
C12
Glu
C14
C16OH
Met
esterifed
C4DC
C5
C0
C16:1OH
C6:1
C18:1OH
C8:1
C16:1
C5OH
High
Low
1.2 1.4 1.6 1.8 2.0 2.2 2.4
VIP scores
E
NPN
F
M
Tyr
Orn
Val
C6:1
C18:1OH
Arg
C18OH
Glu
Gly
ArgSuc
C16:1OH
C4
Met
C2
C4OH
1.0 1.2 1.4 1.6 1.8 2.0 2.2 2.4
VIP scores
F
PN
F
M
esterified/fre
C18:1
C14
C12
C18OH
Ala
C14OH
C3DC
Phe
C6DC
C5OH
C10:1
Arg
C0
C16:1
High
Low
1 2 3 4
VIP scores

Supplementary Figure S2. Metabolites which differentiate PN and no-PN fed groups in **A)** PN fed female VPI cohort vs no-PN group; **B)** PN fed MLPI female cohort vs no-PN group; **C)** PN fed male VPI cohort vs no-PN group; **D)** PN fed male MLPI cohort vs no-PN group.
Discriminant metabolites between PN fed (**E**) and no-PN (**F**) females and males. Each panel reported the top 15 important discriminant features identified by PLS-DA according to the VIP score. The concentrations of the metabolites were log(2) transformed, and Pareto scaled. The intensity of the coloured boxes indicates the relative metabolite abundance in each group.

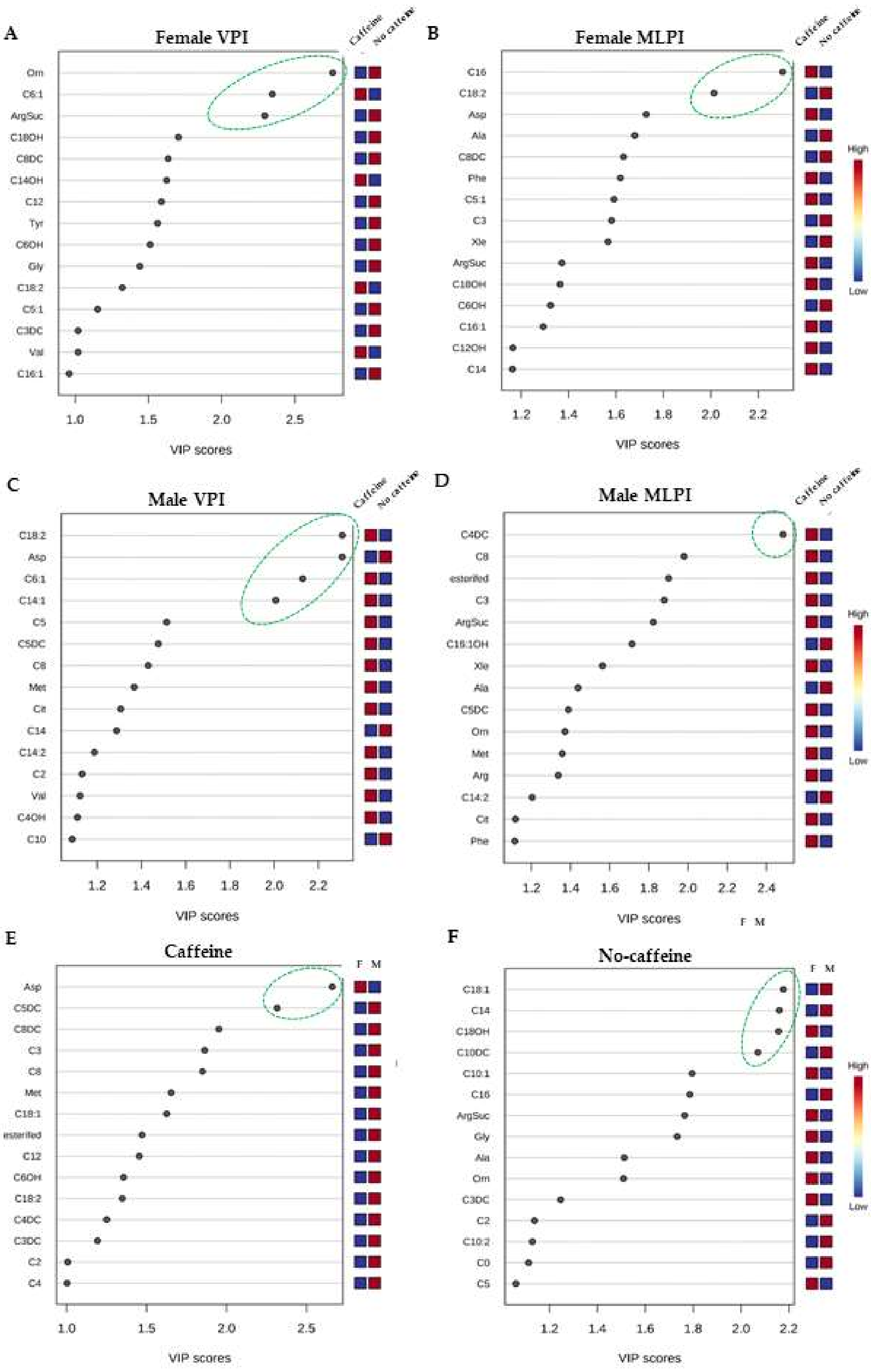

Supplementary Figure S3. Metabolites which differentiate caffeine and no-caffeine groups in **A)** female VPI cohort; **B)** MLPI female cohort; **C)** male VPI cohort; **D)** MLPI cohort.
Discriminant metabolites between caffeine treated (**E**) and no-caffeine treated (**F**) females and males. Each panel reported the top 15 important discriminant features identified by PLS-DA according to the VIP score. The concentrations of the metabolites were log(2) transformed, and Pareto scaled. The intensity of the colored boxes indicates the relative metabolite abundance in each group.
